# Emerging concepts and shifting paradigms for understanding the microbial basis of inflammatory bowel diseases

**DOI:** 10.1172/JCI193969

**Published:** 2025-09-02

**Authors:** Megan S. Kennedy, Eugene B. Chang

**Affiliations:** Department of Medicine, University of Chicago, Chicago, Illinois, USA.

## Abstract

Inflammatory bowel diseases (IBDs) are complex immune disorders that arise at the intersection of genetic susceptibility, environmental exposures, and dysbiosis of the gut microbiota. Our understanding of the role of the microbiome in IBD has greatly expanded over the past few decades, although efforts to translate this knowledge into precision microbiome-based interventions for the prevention and management of disease have thus far met limited success. Here we survey and synthesize recent primary research in order to propose an updated conceptual framework for the role of the microbiome in IBD. We argue that accounting for gut microbiome context — elements such disease regionality, phase of disease, diet, medication use, and patient lifestyle — is essential for the development of a clear and mechanistic understanding of the microbiome’s contribution to pathogenesis or health. Armed with better mechanistic and contextual understanding, we will be better prepared to translate this knowledge into effective and precise strategies for microbiome restitution.

## Introduction

Inflammatory bowel diseases (IBDs) encompass a range of often debilitating chronic gastrointestinal (GI) diseases that impose immense quality-of-life and health-care costs on individuals and society (1). It is increasingly appreciated that among other factors like genetic predisposition and environmental exposures, the gut microbiome plays an essential role in the pathophysiology of IBD. For instance, studies in germ-free mice have demonstrated that gut microbes are required for the development of colitis in several models of genetic susceptibility (2–4) and that transplant of an inflamed gut microbiome into an otherwise healthy gut can induce inflammation (5–7). An abundance of human studies have identified correlations between IBD status and various microbiome omics profiles (8, 9). However, we still lack effective microbiome-based interventions (MBIs) for prevention, management, and treatment of IBD, in spite of the microbiome’s clear contributory role in the etiopathogenesis of IBD.

**Figure 1:**
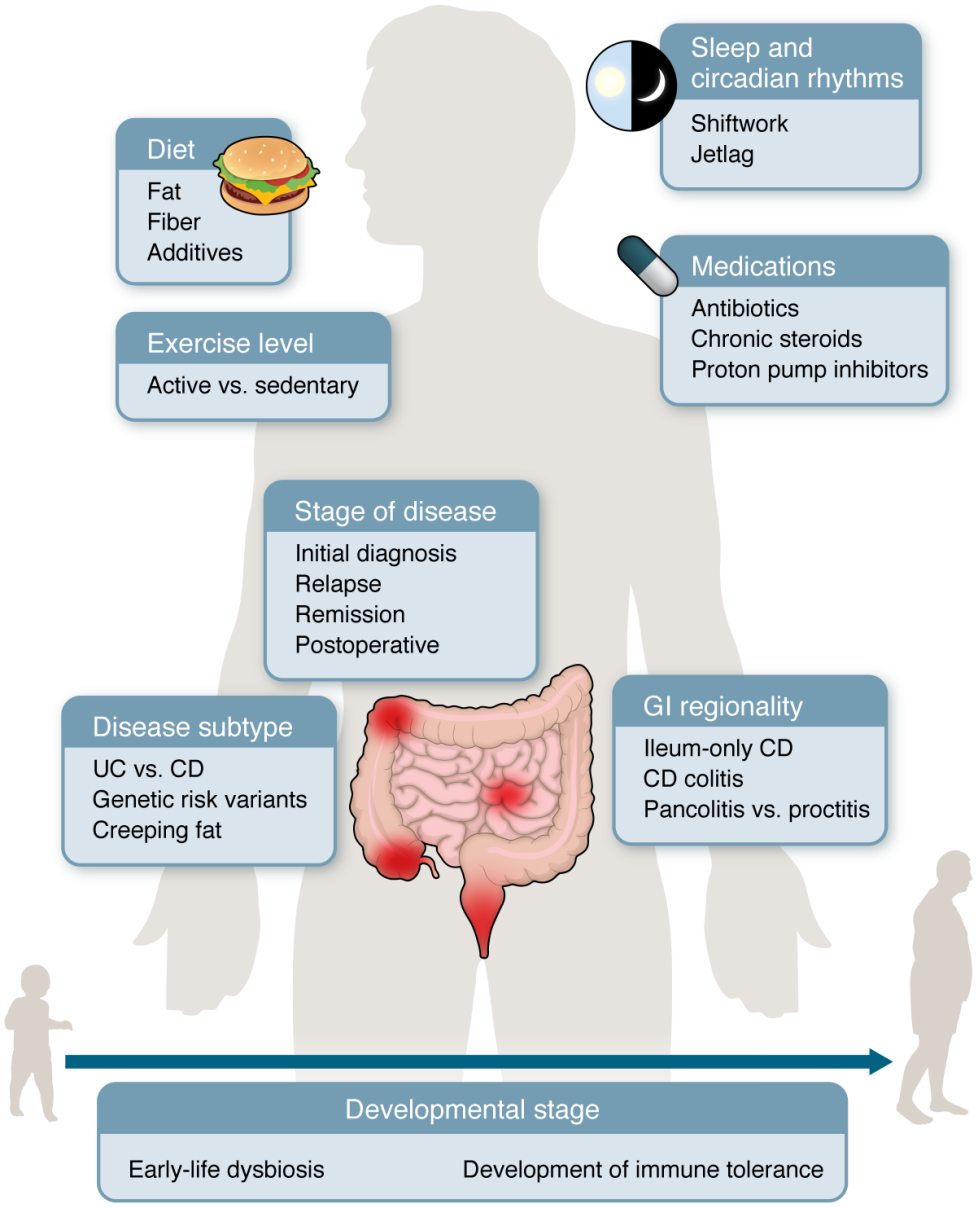
Contextual drivers of the microbiome in IBD. The specific role played by the microbiome in IBD varies across a wide variety of contextual factors. Sleep and circadian rhythms impact the rhythmicity and functional output of the microbiota. Diet affects composition and function of the microbiota. Medications can affect microbiome composition, and the microbiota can reciprocally impact drug metabolism. Across stages of disease from diagnosis to relapse, microbiota composition varies in ways that may help to predict disease activity. Different IBD subtypes are associated with distinct microbiome signature. Distinct regions of the GI tract harbor distinct microbiota profiles both in health and in disease. Exercise and lifestyle impact the microbiota both directly and via effects on motility of the GI tract. Early-life exposures may alter tolerance to commensal microbes, and even the healthy microbiota varies over the human lifespan.

In this Review, we draw on recent primary evidence to formulate an updated conceptual framework for the role of the microbiome in IBD. Although the gut virome and mycobiome, the respective viral and fungal counterparts to the bacterial microbiome, have also recently been highlighted as important contributors to the complex dynamics of the gut ecosystem (10, 11), here we focus on the more extensively characterized bacterial component. In particular, we argue that in order to effectively harness the bacterial microbiome for therapeutic gain, we must take into consideration the basic contextual drivers of gut microbial health and function — that is, the shifting conditions of regional gut ecosystems across distinct phases of disease, diets, microbial network structures, comorbid conditions, medications, and patient lifestyles (Figure 1).

This Review is divided into two main sections: First, we discuss what is currently known about the role of the microbiome in IBD, and how accounting for various aspects of microbiome context can refine that understanding. Second, we discuss existing MBIs and how more deliberate control over microbiome context might improve their efficacy. With this approach, we acknowledge and embrace the complexity of the host-microbe ecosystem as it applies to this multifactorial chronic disease, mining that complexity for meaningful ways to stratify subjects and inform interventional strategies. In so doing, we hope to inspire more incisive research that will bring MBIs into an era of clinical utility.

## Role of the microbiome in IBD across contexts

### Current understanding of the microbiome in IBD.

IBDs, comprising ulcerative colitis (UC) and Crohn’s disease (CD), are complex immune disorders that arise from genetic susceptibility; environmental exposures like diet, toxins, infectious stimuli, etc.; and an imbalance or disruption in the composition and function of the gut microbiome (dysbiosis) — each necessary, but not sufficient, to cause disease. For example, a patient whose immune system is genetically programmed to respond at a lower stimulus threshold might mount a proinflammatory response against a benign ingested or microbial antigen passing through the GI tract (12). In patients with CD, this hyperresponsiveness could be caused by mutations in genes like NOD2, which is involved in intracellular processing of bacterial components (13, 14), or ATG16L1 or IRGM, which are involved in autophagy and clearance of intracellular pathogens (15). In UC or CD, various mutations in IL-23 pathway genes, including IL23R, IL12B, STAT3, and JAK2, serve to induce a hyperactive Th17 cell response (16). The resultant inflammation may damage the epithelial barrier, allowing the commensal microbes to penetrate the gut mucosa and come into contact with deeper tissues of the GI tract that are laden with immune cells (17).

Importantly, the microbes that trigger the immune system need not be the initiating cause of inflammation, nor do they even need to be strictly pathogenic organisms (18). The once-predominant view that a single pathogenic microbe could be isolated as the causative agent of disease has been almost wholly unsupported in the IBD-microbiome literature (19). Study after study has failed to consistently identify a single causative agent in patients with active IBD, and even in the handful of studies that could link one organism to disease (20–22), these organisms more often take the form of opportunistic “pathobionts,” or otherwise commensal organisms that become pathogenic only under specific disease-promoting conditions (18, 23).

Rather, an extensive body of literature now shows that the gut microbiome of IBD patients often has few unique community members compared with that of healthy control individuals, but instead exhibits skewed proportions of the same “healthy” taxa, resulting in altered overall community composition and function (19, 24). Thus, microbiome “eubiosis” or “dysbiosis” can be better understood as emergent properties of the community as a whole, rather than the work of individual “good” or “bad” microbes.

How exactly a healthy microbiome transitions toward dysbiosis, and whether that is cause or consequence of inflammation, remains the subject of intense research and debate (19, 25). In all likelihood, the answer depends on *context*. Genetics, environment, and microbiome interact in a complex web of feedback responses to tip the balance toward health or inflammation. An otherwise benign antigen (26) or commensal microbe (27, 28) might trigger a genetically hyperresponsive immune system. Lifestyle factors like diet or cigarette smoking might shift the microbiome toward dysbiosis that predisposes even a stable immune system to inflammation (29, 30). The task at hand is to identify which elements of microbiome context best enable us to understand and manage disease or to maintain states of remission.

### Disease subtype context: the wide range of IBDs.

A diverse array of semi-overlapping disease presentations fall under the broad umbrella of IBD. Most commonly, these are subcategorized into UC or CD. The microbiome profile of patients with either subtype is altered compared with that of healthy control individuals, with IBD patients exhibiting reduced diversity; increased levels of Enterobacteriaceae; loss of putatively beneficial taxa like Ruminococcaceae and Lachnospiraceae; and an altered metagenome and metabolome with enrichment of sphingolipids and primary bile acids (8, 31–33). But depending on which GI tissues are involved, the microbiota of UC and CD are also distinguishable from each other (31, 34), to the extent that disease subtype can be accurately predicted from microbiome signature alone (35).

Even within these major branches of IBD, however, there is significant variability in disease presentation, prompting efforts to further subclassify and stratify patients. For CD, subclasses have been proposed along lines of disease behavior (perforating, stricturing, creeping fat, etc.) (36), age of onset (36, 37), disease localization (ileal, colonic, ileocolonic, perianal, perioral, etc.) (36, 38), immune cell profiles (26, 39), molecular markers (40), and genetic risk variants (41, 42). UC has similarly been subtyped by localization (proctitis, left-sided UC, pancolitis) (43, 44), disease severity (45), genetic factors (41), and immune profile (46–48). These factors could all be reasonably expected to modify the physical and immunological environment experienced by the gut microbiota. Indeed, the microbiota of healthy individuals is known to vary by GI region (49), age (50), genetics (51), and immune profile (52). Correspondingly, many subtyping schemes have been shown to correlate with distinct microbiota states, likely reflecting shared underlying pathophysiology that can help us to more effectively deduce mechanisms of disease.

An excellent example is CD with or without creeping fat. “Creeping fat” refers to hyperplastic mesenteric adipose tissue that wraps around inflamed intestinal lesions in CD, particularly in the antimesenteric region, worsening inflammation, inducing fibrosis, and promoting the development of strictures that predispose patients to bowel obstruction (53). Researchers determined that individuals with creeping fat exhibited a distinct microbiome localization signature — that is, in patients with creeping fat, members of the microbiome migrate via transmural lesions of the bowel wall into the surrounding fatty tissues (54). Once in the mesenteric adipose, these microbes interface with the immune system to induce inflammation, tissue remodeling, hyperplasia, and fibrosis (55). Thus, for this disease subtype, a precise mechanism was traced from aberrant microbiome localization to disease manifestation.

Other schemes of disease subclassification have similarly shown correlations with microbiome state. For example, Moustafa et al. identified a modest correlation between IBD genetic risk variants and microbiome composition (56), and Imhann et al. showed that even healthy individuals with IBD genetic risk factors have a microbiome distinct from those without genetic risk factors (57). Ghosh et al. identified distinct microbiome signatures for IBD among different age groups (58), Kedia et al. showed that the UC microbiome varies across categories of disease severity (59), and multiple groups have shown associations between CD flare localization and microbiota composition (38, 60). Additional experimental follow-up is needed to transition from correlative findings to mechanistic understanding of the microbiome’s role in these scenarios.

### GI regional context: biogeography of the bowel.

Regarding methods of subclassifying UC and CD, there is mounting evidence in support of stratifying patients according to which GI tissues are affected by inflammation, particularly for CD, which spans a greater diversity of tissues than UC (38, 61). It has been well established that the gut environment varies along the rostro-caudal axis anatomically and physiologically, with biochemical gradients in factors like pH (62), oxygen level (63), mucus thickness (64), and bile acid pool (65). The immune system is likewise distinct across regions of the GI tract that serve different physiological purposes (66). In healthy individuals, these factors all contribute to differences in microbial biomass, composition, and function across each region of the gut (49, 67).

It is hardly surprising, then, that disease location impacts microbiome signature and that patients with ileum-only versus colon-only CD exhibit distinct microbiome profiles (38, 60, 68). Whether cause or consequence of inflammation and immune activation, the pool of microbes available to participate in pathologic processes is distinct across GI regions, and therefore we can expect that GI region will be a pivotal factor in determining microbial involvement in disease. This premise is supported by recent work that combined host and microbiome multiomics data, and found that the multiomics profile of colon-only CD is more similar to that of UC than to ileal CD, with relative enrichment of neutrophil-related proteins and the strict anaerobe *Bacteroides vulgatus* in the colon (60). These similarities across colonic CD and UC could suggest a convergent microbial response to inflammatory conditions in the colon. More fundamentally, these data illustrate the importance of evaluating region-specific disease presentations separately.

An important corollary of the notion that GI regional context matters for understanding microbial drivers of disease is that study of fecal samples is not sufficient to gain insight into local disease processes in other parts of the GI tract. Microbial activity in the small bowel is simply not captured by fecal samples (69), and although the duodenum, terminal ileum, and colon can be reached and sampled via labor-intensive endoscopic approaches, the remaining approximately 20 feet of small bowel is largely inaccessible. Tools like CapScan or the SIMBA Capsule, ingestible pill-shaped sampling devices, are in development to enable relatively noninvasive luminal sampling throughout the upper and lower GI tract (70, 71). Several studies have shown that even within the lower GI tract, the fecal microbiome is distinct from the microbiome of mucosal scrapings or luminal contents of the distal colon (72, 73). Thus, researchers must carefully weigh the costs and benefits of mucosal versus fecal sampling when designing studies.

### Temporal context: initial presentation, remission, and relapse.

IBDs are fundamentally characterized as chronic, relapsing and remitting diseases. Much of the work evaluating the multifactorial causes of IBD has relied on cross-sectional studies comparing healthy control with affected individuals. However, this approach limits our ability to infer time-course dynamics, and inappropriate aggregation of data across phases of disease may obscure microbial signatures associated with the onset of inflammation.

Because we cannot yet predict when IBD will initially present, longitudinal cohort studies with true pre- and postdiagnosis samples are rare. These prospective studies typically enroll unaffected relatives of patients with IBD, given their high risk for eventually developing IBD (74, 75). In one such study with pre- and postdiagnosis samples from patients with UC, researchers found that although major reshaping of the microbiome happens after diagnosis, subclinical changes in gut microbiome composition, metagenome, and proteolytic activity do precede disease onset. Follow-up experiments in germ-free mice showed that the pre-UC microbiota, but not that of healthy control individuals, was sufficient to increase proteolytic activity and increase the abundance of polymorphonuclear immune cells. Together, these results suggest that while the microbiota responds robustly to inflammation, it may also play an early role in causing inflammation.

Another approach to resolving the temporal dynamics of the microbiome in IBD is to compare samples taken from the same patients during active disease and during remission. Several studies now suggest that the microbiome of patients with IBD generally fluctuates more over time than that of healthy control individuals (31, 76, 77), although there is no clear consensus regarding whether specific microbiome compositional shifts precede or correlate with states of disease flare or remission (78, 79). A notable shortcoming of these studies is the variable (and poorly reported) number of days between “remission” and “active disease” sampling. Among the studies reviewed here, the shortest reported interval between remission and flare samples was one month (31). Given that microbiome composition can shift on a timescale of days to weeks in response to host and environmental stimuli (80), sampling on a timescale of months may not adequately capture the dynamics that precede and contribute to disease, especially given the particularly dynamic nature of the IBD microbiome.

Patients who undergo bowel resection for refractory IBD provide unique insight into the dynamics of disease recurrence. After surgical removal of inflamed bowel, “clean slate” samples are taken from the remaining healthy tissues and are compared with inflamed tissues as disease recurs. For CD, the postoperative, healthy ileal microbiota of individuals whose disease eventually recurred is distinct from the microbiota of those who maintain remission (81–83). Several such studies independently found that, at the postoperative time point, those who went on to develop recurrence had relative increases in the abundance of facultative anaerobes like *Fusobacterium* and various Proteobacteria genera, with losses of butyrate-producing taxa from the Lachnospiraceae or Ruminococcaceae families (81–83).

For UC, postoperative recurrence has been studied with the pouchitis model: after total colectomy with ileal pouch anal anastomosis, nearly 50% of individuals will eventually experience UC-like inflammation in the previously unaffected ileal tissue of the J-pouch (84). In the healthy pouch, specific, distinct microbiome changes have been documented among those who go on to develop pouchitis compared with those who do not, including overall loss of diversity and changes in abundances of specific anaerobes (85–87).

Collectively, these time course studies indicate that although the microbiome certainly responds to inflammatory processes and abrupt changes in host physiology like those encountered after surgery, microbiome changes themselves may precede, perpetuate, or exacerbate inflammation in a feedback cycle with the host and immune system. That is, microbial dysbiosis can be both cause and consequence of inflammation.

### Developmental context: consequences of early-life exposures.

On a broader timescale, recent evidence suggests that IBD pathophysiology and corresponding microbiome changes may depend on developmental cues from early life. Multiple mouse studies show that after antibiotic treatment of pregnant dams, the dysbiotic microbiome inherited by offspring predisposes them to spontaneous colitis later in life (88, 89). Miyoshi et al. further demonstrated that early-life exposure to a diverse array of microbes promoted the development of immune cell subsets that imparted tolerance to those microbes upon reexposure, whereas mice that inherited a low-diversity, dysbiotic microbiota developed inflammation when first exposed to those otherwise benign commensal taxa at a later stage in life (88). Similar results were identified in humans by Gevers et al., who found that early-life exposure to antibiotics amplified CD-associated microbiome dysbiosis (90). Thus, for autoimmune pathologies like IBD, understanding not only the peri-disease microbiome but also the broader history of the microbiome may shed light on disease pathogenesis.

### Lifestyle context: diet, medications, and sleep.

Perhaps the most expansive and daunting aspect of microbiome context that is nevertheless essential to understanding the role of the microbiome in IBD is host lifestyle. The bacteria that colonize the human gut can adapt and evolve in response to new stimuli on an extraordinarily rapid timescale (91, 92). This means that behavioral changes in factors like diet (80, 92), medication use (93), sleep habits (94, 95), and exercise (96) can have a nearly immediate impact on microbiome community composition and function. When ignored, these factors will undoubtedly confound our search for microbial determinants of disease. However, when properly accounted for, they may even may even be exploited as some of the lowest-risk, least-invasive, and least-expensive approaches to modifying the course of disease via the microbiome.

Among the most salient lifestyle drivers of microbiome change in IBD is diet. The microbiome responds robustly to changes in diet, with more recent work identifying specific responses to dietary components like saturated versus unsaturated fats, preservatives, processed sugars, and specific fiber sources (or their absence) (97–99). For example, several murine studies have shown that a high-fat diet can induce proinflammatory dysbiosis (100, 101). The role of fiber and complex carbohydrates in protecting against inflammation is also increasingly appreciated: work from Desai et al. showed that in the absence of microbiota-accessible carbohydrates, microbes preferentially consume host-derived mucosal glycoproteins, invading and thinning the protective mucosal barrier in the colon and predisposing to inflammation (102).

On a molecular level, the byproducts of microbial metabolism, which depend profoundly on host diet, can also interface directly with the immune system to modulate inflammation. For instance, short-chain fatty acids (SCFAs) like acetate, propionate, and butyrate are produced by microbial fermentation of dietary fiber. These compounds, and especially butyrate, can directly act via G protein–coupled receptors GPCR41 and GPCR43 to promote the differentiation of antiinflammatory regulatory T cells (103). Low-fiber diets with correspondingly low levels of butyrate have been linked to the development of IBD (104). Similarly, diet impacts host production and circulation of primary bile acids, which serve to aid in the digestion and absorption of dietary fat. Secondary bile acids are produced exclusively by bacterial transformation of primary bile acids, and beyond their role in lipid absorption can act via the FXR and TGR5 receptors to modify intestinal permeability and barrier integrity (105) as well as to directly stimulate antigen-presenting cells (106). Imbalances in bile acid profile have been associated with the development of both UC and CD (8, 106, 107). Indole metabolites resulting from microbial transformation of dietary tryptophan, by contrast, are protective against inflammation (108).

Medication use is another lifestyle factor with profound influence on the microbiome and predisposition to IBD. Antibiotics, for example, have played a complex role in IBD management. On one hand, antibiotic treatment has been therapeutic for some cases of IBD and is first-line for pouchitis (109). On the other hand, antibiotic-induced dysbiosis is a risk factor for the development of IBD, particularly with antibiotics are used in multiple times early in life (110–112). Mainstays of IBD treatment like corticosteroids have also been shown to impact the colonic microenvironment and the gut microbiota in murine models (113). Other nonantibiotic medications including proton pump inhibitors and metformin have well-documented effects on microbiome composition and function (114–116). Thus, IBD patients whose symptom management or comorbidities require a broad and multimodal treatment regimen may ultimately be at greater risk of microbiome dysbiosis and disease recurrence as a consequence of those very interventions.

Other lifestyle factors like sleep (117) and circadian rhythms (94, 95) or exercise and general activity level (118) affect gut function and motility both directly and via the microbiome. There are nearly countless ways that host lifestyle choices could affect the microbiome. In addition to continuing dedicated exploration of the potentially causal effects of these factors on IBD pathogenesis via the microbiome, future work must at minimum collect appropriate metadata on lifestyle elements like diet, medication use, activity level, and sleep behaviors so that their impacts can be analytically disentangled and accounted for.

## Utilizing microbiome context to improve MBIs

### Quantifying success: from molecular change to clinical outcomes.

**Figure 2:**
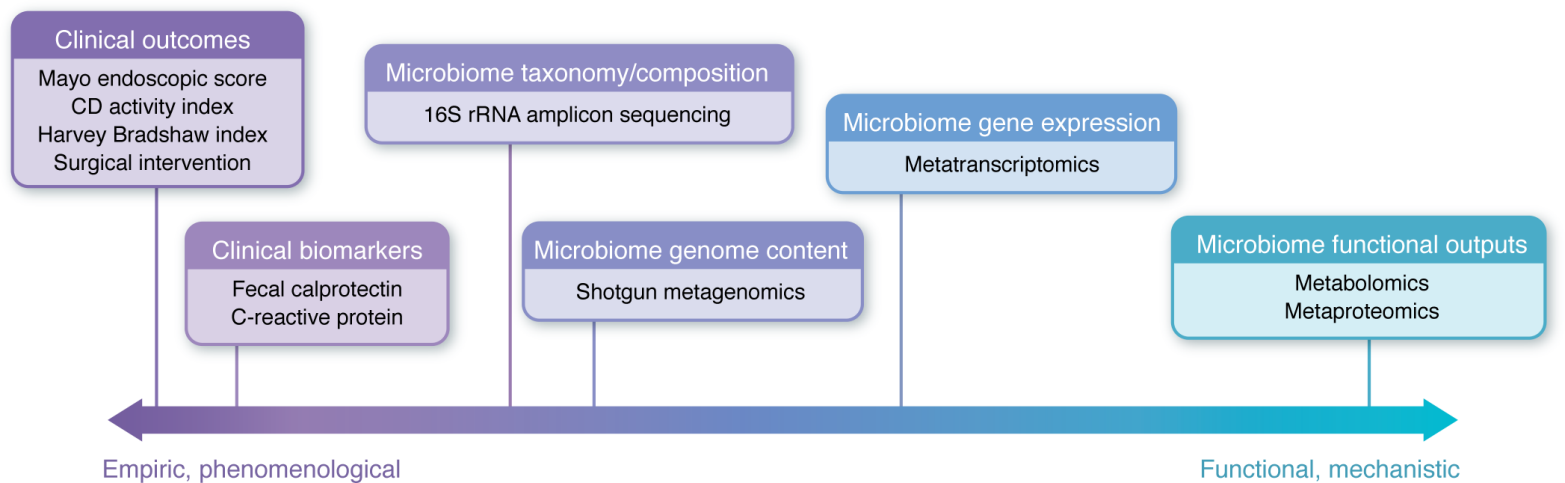
Metrics to quantify the success of microbiome-based interventions. Methods of quantifying the impact of MBIs span diverse metrics ranging from empiric clinical outcomes to quantification of the microbiome itself to the evaluation of the functional outputs of the microbiome. Clinical outcomes like the Mayo endoscopic score for UC or the CD activity index are used to gauge disease activity based on endoscopic findings and clinical symptomatology. Clinical biomarkers like fecal calprotectin and serum C-reactive protein provide less-invasive objective measures of inflammation severity. Fecal 16S rRNA sequencing provides a low-resolution readout of microbiome composition, while shotgun metagenomics evaluates the genomic content and functional capacity of a sample, and metatranscriptomics quantifies mRNA to quantify gene expression and evaluate functional activity of the microbiota. Metabolomics and metaproteomics evaluate the functional outputs of the gut microbiome (i.e., metabolic byproducts and proteins produced), which can often directly interact with the host. These strategies each address different elements of the IBD phenotype and are often most informative when evaluated together.

First and foremost, a discussion of how to quantify successful use of MBIs in the treatment of IBD is warranted (Figure 2). The vast majority of clinical research employing MBIs for the treatment of IBD evaluates exclusively clinical outcomes: disease remission or symptom improvement (119). Of course, clinical outcomes are most important for patients, but empiric clinical measures alone leave little room to evaluate subclinical microbiome change resulting from an intervention. Many studies have at least perfunctorily examined microbiome composition via 16S sequencing. However, mechanistic work in preclinical models has increasingly shown that taxonomic data may have limited value in predicting the functionality of a microbial community, as there is extensive functional redundancy among taxonomically disparate microbes (120, 121).

Many researchers have recently turned toward downstream functional metrics of microbiome community function like metabolomics or metaproteomics (122, 123). Products of microbial metabolism like SCFAs, conjugated bile acids, tryptophan metabolites, and various amino acids have been proposed as salient readouts of microbiome function (124–128). Thus, with the establishment of healthy reference ranges for these metabolites, they could provide clues into specific mechanistic shortcomings of the microbiome and, perhaps, how to fix them.

Importantly, even reliable, mechanistically informed metrics of microbiome functional change may not shift immediately or completely following intervention. Although the microbiome can respond very rapidly to intervention, pursuing the right *trajectory* of microbiome change may require successive or iterative cycles of intervention. Thus, to monitor the success of MBIs, we may require repeat, quantitative measurements over time, much in the way trends in standard blood chemistry tests like a complete blood count or basic metabolic panel are used in clinical practice (124). This can help us to identify scenarios in which subtle microbiome change may precede macro-scale clinical change.

With better metrics for quantifying the impact of MBIs across scales from molecular change to clinical response, we can more effectively connect the mechanistic dots between intervention and outcome.

### Strategies for microbiome modulation: ablation, replacement, and support.

**Figure 3:**
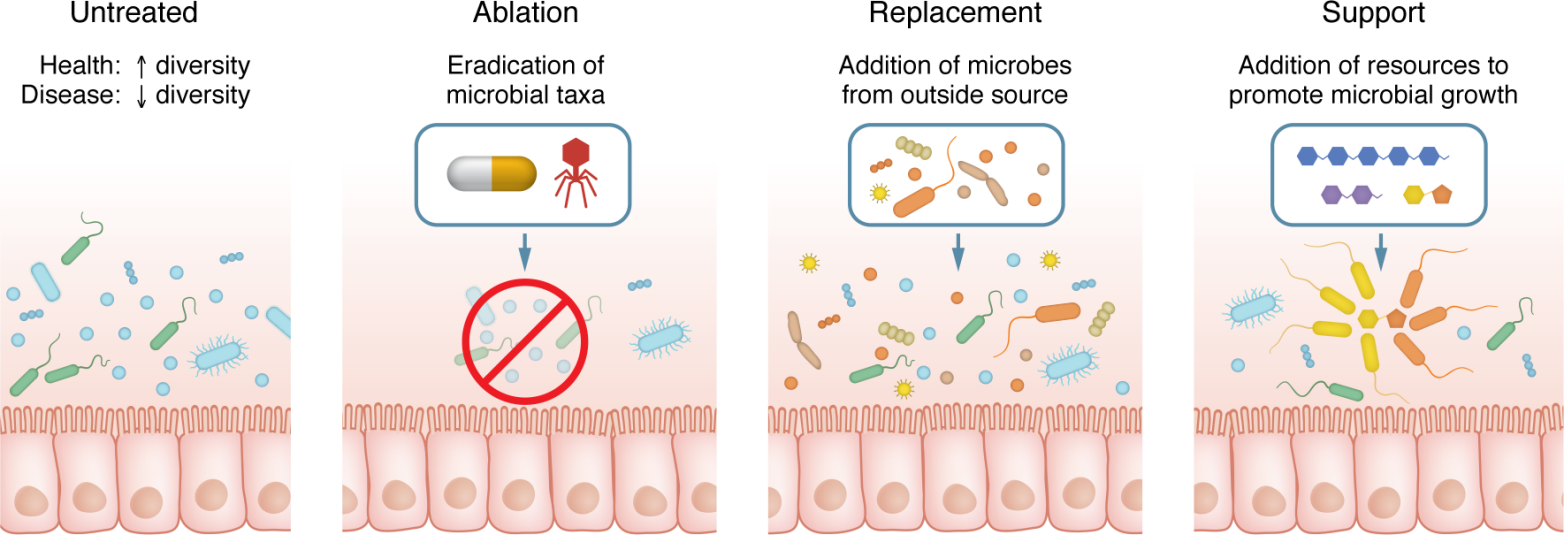
Strategies for MBIs. The “Untreated” panel schematically depicts a microbial community. “Ablation” refers to the eradication of the endogenous microbiome via methods like antibiotic treatment or via more targeted approaches like phage therapy. “Replacement” refers to strategies that introduce exogenous microbes into the gut, such as probiotics, synbiotics, FMT, or live biotherapeutics. “Support” represents strategies that involve changing the resource environment to support microbial growth, such as dietary interventions or prebiotics, which provide fermentable fiber sources that are selectively metabolized by gut microbes.

Current strategies for microbiome manipulation can be broadly characterized as microbiome ablation, replacement, or support (Figure 3 and Table 1).

Antibiotics differentially ablate members of the native microbiome. Their impact can vary from person to person (129) based on factors like initial microbiome composition (130), treatment duration, and dosage (131) or confounding elements like host diet (132), and they provide no subsequent intervention to guide recovery. In the context of IBD, antibiotic use is limited primarily to the management of infectious complications (e.g., abscess, fistula, bowel perforation, or perioperative) (133–135) and pouchitis (109). Other promising ablative strategies under development include viral phage therapy, in which bacteriophages are engineered to target specific bacterial taxa and introduced into the gut for precision removal of undesired microbes (136, 137).

Among existing MBIs, microbiome replacement or transplant strategies have commanded the vast majority of clinical, academic, and commercial interest. Replacement approaches encompass probiotics, synbiotics, fecal microbiota transplant (FMT), and live biotherapeutics (Table 1) and generally aim to replace “disease-promoting” microbes with different, putatively “health-promoting” taxa. Probiotics are live bacteria most often derived from fermented food products. These bacteria are typically not well-adapted to survive in the human gut and have shown limited ability to engraft in their recipient community (138, 139) or shape a sustainable trajectory of microbiome change (140). “Synbiotics” denotes a pairing of probiotic and resource supplement to promote the growth of that probiotic (141). This premise is rational, and there is evidence supporting the idea that specifically tailored metabolic support can promote the growth and engraftment of exogenous microbes (142), but in practice, most synbiotics are composed of fermented food–derived probiotics with a carbohydrate resource that is not selectively metabolized by the probiotic over other members of the endogenous microbiota (141).

FMT and live biotherapeutics transition away from food-derived microbes and instead introduce taxa derived from a human gut. FMT refers to the bulk transplant of stool from a healthy human donor into an unhealthy individual. “Live biotherapeutic products” is a new term developed by the US Food and Drug Administration to categorize products containing live organisms like bacteria that have proven clinical efficacy in treating disease, and they are regulated as drugs (143). Although FMT is very effective for the treatment of refractory *Clostridioides* *difficile* infection (rCDI) (144), its success rate in IBD has been inconsistent, hovering near 40% under most protocols (145). Only two live biotherapeutic products are currently approved in the US, both for the treatment of rCDI (146, 147) and each derived from a human fecal community. Three new live biotherapeutic products are in phase II clinical trials for the treatment of CD and UC (148–150).

Finally, interventions to support microbial growth by changing the gut resource environment include prebiotics and dietary interventions. Prebiotics seek to guide microbiome change by providing resources selectively utilized by gut microbes in order to support their growth (151). Most prebiotics are made of compounds like inulin or fructooligosaccharides, which are carbohydrate compounds that can be fermented into SCFAs by a broad range of bacteria, but not the host (151, 152). While they may generally support the growth of commensal bacterial, there is little evidence to suggest that they promote compositional shifts or supplement the growth of specific, potentially beneficial taxa. Although dietary change can drastically affect the microbiota, it is usually relatively untargeted, and adherence can be difficult for patients.

“Postbiotics” refers to downstream products of microbial metabolism or components of microbes themselves, which are introduced into the gut without any live organisms. These are used not so much as a method of microbiome change as a means of sidestepping the microbiome altogether by introducing microbially derived molecules that can act directly on the host (153). Interestingly, early evidence from experiments using postbiotics have shown that these products can nevertheless act to shift microbiome composition and function (154, 155).

Each of these strategies derives from reasonable hypotheses about how to enact microbiome change. However, the specific details of their execution, especially with regard to microbiome context, must be examined to improve their efficacy.

### Future directions: embracing context to improve MBIs.

Fundamentally, most existing MBIs neglect microbiome context and have been consequently plagued by inconsistency (156, 157). Microbiome ablation strategies like antibiotics, for instance, rarely take into account host diet, which can fundamentally alter the trajectory of community re-assembly following a perturbation (132). Studies of prebiotics and synbiotics fail to determine whether resource supplements will selectively promote the growth of one or multiple endogenous microbes versus an introduced probiotic. FMTs are conducted without standardized administration protocols (158, 159), without consideration of where inflammation is most severe, which microbes can thrive in that region and whether they are present in the transplanted community, and without any real characterization of host and recipient communities (160).

These problems can be addressed through rationally designed intervention in which a specific goal is approached via a tailored strategy, taking into account contextual information as available. For instance, if we would like to address microbial dysbiosis in the small bowel, we should consider MBIs that promote small bowel microbiota (161). If we would like to promote the growth of specific microbial taxa, we should identify those taxa’s metabolic needs and target them specifically and selectively through prebiotics or dietary change (142, 162). If we would like a transplanted microbial community to serve a particular function, we should either formulate a synthetic community to achieve those functional goals or at least screen donor communities, bioinformatically or in vitro, for functional criteria. We should moreover carefully consider how that community might change under the dietary or inflammatory conditions of the recipient, and in competition with the recipient microbiome. This could guide decisions about recipient diet or supplementation, optimal timing of MBI relative to disease flare and immunomodulatory interventions, and whether antibiotic pretreatment of the recipient may be of benefit.

Given the temporal context of microbial community change and disease course, effective MBIs may need to span multiple rounds of targeted interventions. In macroecology, “secondary succession” refers to a process of gradual community change as the ecosystem undergoes iterative cycles of environmental change and community adaptation and response (163). For instance, dysbiotic conditions after a disturbance like a forest fire permit the growth of only tough, weedy grasses and pioneer species; but these species modify the soil in a way that allows the growth of bushes and shrubs, which change the environment to enable the growth of larger trees, in a process that progresses step by step until the climax community is achieved (164). Microbiome transplant after a disturbance like the severe inflammation of IBD may successfully introduce some pioneer taxa, but the gut environment has not yet changed enough to accommodate the growth of the climax community. Repeat transplant of whole communites or staged introduction of specific taxa or metabolites, chosen based on quantitative metrics of gut environmental recovery, facilitate a successional process of microbiome reconstitution.

Precision medicine strategies are emerging as a way to integrate individualized contextual data to generate personalized therapeutic interventions (165). For instance, one study developed a machine-learning tool that used individual data on blood markers, dietary habits, anthropometric measurements, physical activity, and gut microbiota to produce personalized dietary recommendations for better glycemic control (166, 167). In the context of IBD, such approaches could integrate information on genetic susceptibility, disease subtype and localization, medication use, prior antibiotic exposure, diet, and microbiome composition and function to predict features like chance of disease progression, drug response, or even optimal interventions for microbiome modulation. Even without personalizing treatments for each individual with IBD, applying this approach to a sizeable cohort could help to identify salient characteristics of patient subsets who respond similarly to certain interventions.

By embracing as much context as is readily available when designing interventions, MBIs will become more precise, consistent, and effective tools in the treatment of IBD.

### Multimodal approaches to IBD management.

IBD pathogenesis results from the dysregulation of many factors, including the microbiome, the spatial and structural physiology of the epithelium and mucosal barrier, environmental metabolites and oxygen levels, and the immune response. The current standard of care for IBD medical management relies on biologic therapies that target only the immune system, which integrates cues from all these elements (168). There has been minimal research on multimodal approaches that combine, for instance, microbiome interventions, dietary interventions, wound-healing and mucosal-protective strategies, and immunosuppressive therapies. By simultaneously targeting multiple aspects of IBD pathophysiology, we might more effectively be able to tip the scales in the direction of recovery.

More specifically, inflammation itself strongly affects microbiome composition and function (169). Attempting to modify the microbiome without addressing a florid inflammatory response is unlikely to bear fruit, as a transplanted microbiome may itself be changed by the environmental inflammation. Similarly, unaddressed metabolic derangements like bile acid dysregulation in CD involving the terminal ileum may hinder attempts to modify the microbiome. Bile acids are directly bactericidal to many taxa (170), and if imbalances in their abundance profile are not addressed, a microbial transplant will experience these bactericidal effects just as the native community did (171). Restoring a balanced bile acid profile by modifying dietary intake could be one way to lessen this impact, in conjunction with other interventions.

Wound healing remains an understudied component of IBD pathogenesis and therapy. If the heightened immune activity of IBD is thought to result in part from direct contact between submucosal immune effectors and microbes at wounded epithelial surfaces, then wound healing should be a critical element of any rational therapeutic approach (172). Strategies to promote wound healing, which to date include methods like mesenchymal stem cell transplant (173) or administration of intestinal growth factors like teduglutide (174), growth hormone (175), or epidermal growth factor (176), could change the spatial ecology of inflammation. Promising new research is exploring the use of synthetic hydrogels to restore the mucus layer and promote wound healing (177). Interestingly, recent data indicate that gut microbes and their mediators are needed for proper intestinal stem cell development, restoration of gut microenvironment, and revascularization of inflamed tissues, paving the way for healing (178–180).

By simultaneously addressing all of these contextual elements, their impacts and benefits might feed back into each other to deescalate the inflammatory cycle.

### Prevention over treatment.

Although we do not yet have strong tools to predict new onset of IBD, better monitoring of microbiome dysfunction, possibly via regular measurement of microbiome functional outputs like the metabolome, could provide a means of identifying early subclinical shifts that precede more overt inflammation. Addressing such concerns before the onset of inflammation may enable treatment and prevention by more cost-effective and less invasive means such as dietary intervention.

## Conclusions

Research into the role of the microbiome in IBD has expanded tremendously over the past several decades, but translation of this body of work into clinically effective therapies has been slow, and at times frustrating. Rather than be discouraged by the fractal nuance of this complex, multi-scale ecosystem, we should be inspired by the opportunities that the microbiome and its drivers can provide as unique avenues of intervention. The details of microbiome context *do* matter to the trillions of microscopic eco-evolutionary agents inhabiting our guts. A contextualized, multi-pronged therapeutic strategy — grounded in ecological and mechanistic insight — may be essential to break the cycle of chronic inflammation in IBD.

## Figures and Tables

**Table 1:**
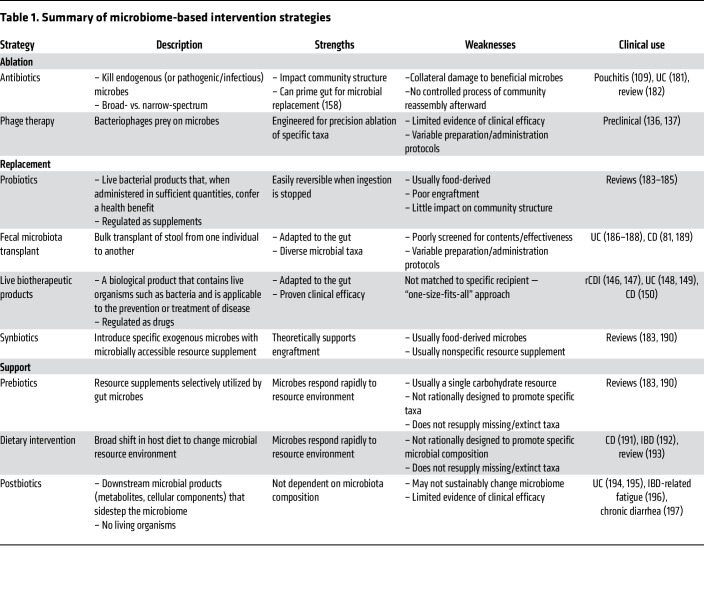
Summary of microbiome-based intervention strategies

## Version 1 09/02/2025

Electronic publication
